# The complete chloroplast genome sequence of *Malus* × *adstringens* Zabel ‘Hopa’ (Rosaceae)

**DOI:** 10.1080/23802359.2023.2292158

**Published:** 2024-01-25

**Authors:** Leiming Dong, Ruizhen Wang, Hengxing Liu, Guowei Xia, Jian Quan, Ling Guo, Minghui Chen

**Affiliations:** a Key Laboratory of National Forestry and Grassland Administration on Plant Ex situ Conservation, Beijing Floriculture Engineering Technology Research Centre, Beijing Botanical Garden, Beijing, China; b College of Eco-Environmental Engineering, Guizhou Minzu University, Guiyang, China

**Keywords:** *Malus*, crabapple, chloroplast genome, phylogeny relationships

## Abstract

*Malus* × *adstringens* Zabel ‘Hopa’ is an important crabapple cultivar with significant ornamental value. Here, we assembled its complete chloroplast (cp) genome using the next-generation sequencing technology to clarify the phylogenetic relationships in *Malus*. The total length of the complete chloroplast genome was 160,230 base pairs (bp) with a GC content of 36.50%, consisting of a large single-copy (LSC) region with a sequence length of 88,310 bp, a small single-copy (SSC) region with a sequence length of 19,196 bp, and a pair of inverted repeat (IR) regions of 26,362 bp. The complete chloroplast genome contained 128 genes, namely 84 protein-coding genes, 36 tRNA genes, and 8 rRNA genes. In addition, 73 SSRs were found in the *M*. ‘Hopa’ cp genome. The phylogenetic relationship of *M.* ‘Hopa’ in *Malus* is closely related to *M. spectabilis* (Aiton) Borkh. and then to *M. sieversii* (Lebed.) M. Roem. Our results demonstrate that it is feasible to resolve the phylogenetic relationships of crabapple cultivars and identify their putative maternal lineages using cp genomic data.

## Introduction

**Figure 1.**
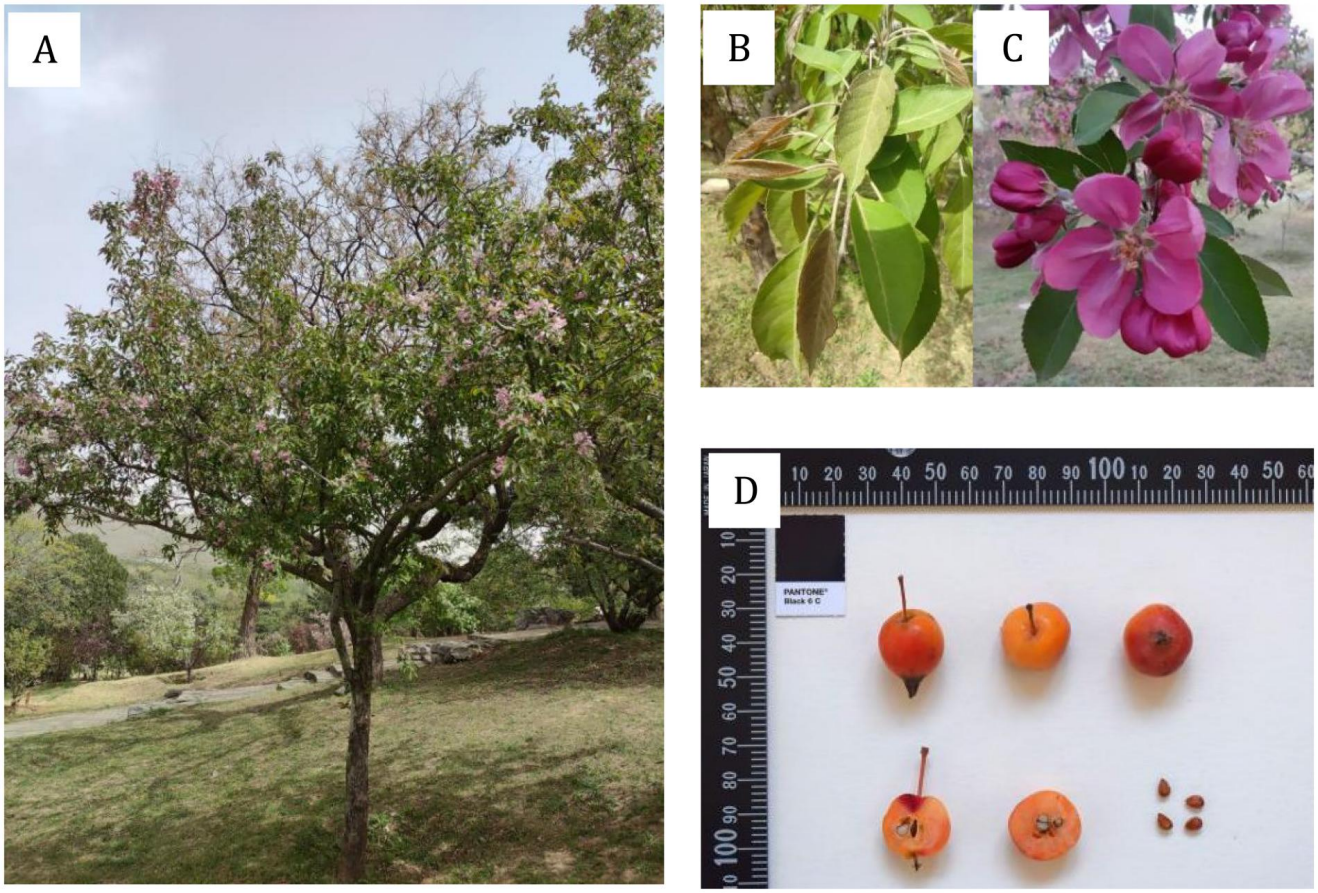
Plant images of *Malus* × *adstringens* ‘Hopa’. A. Individual tree with a height of 2.5 m. B. Leaves. C. Pink and single flowers, 4-5 cm across. D. Bright red fruits with a diameter of 2 cm. This photo was taken by Leiming Dong at Beijing Botanical Garden with the author’s approval for use.

The ornamental crabapples (*Malus* spp., Rosaceae) are woody plants with fruit diameters of less than 5 cm, whose flowers, leaves, fruits and other traits have significant ornamental value (Fiala 2003). *Malus* × *adstringens* Zabel ‘Hopa’ (Hopa) is the first selection of *M. pumila* Mill. ‘Niedzwetzkyana’, probably a cross of *M. pumila* Mill. ‘Niedzwetzkyana’ × *M. baccata* (L.) Borkh. (Fiala 2003), and has been a widely used crabapple cultivar in horticulture (Figure 1). However, Hopa is now less used due to its susceptibility to a fungal disease called scab which causes defoliation and is detrimental to its health and artistic value (Nichols 1985). In addition, the phylogenetic relationships of species and cultivars within the genus *Malus* have long been problematic because of widespread natural or artificial hybridization. Therefore, there is a need to confirm its phylogenetic relationship in the genus *Malus* and to carry out the conservation of genetic resources.

Chloroplasts are maternally inherited in angiosperm plant species, whose genomes have been extensively used to investigate genetic diversity, phylogenetic relationships and to identify germplasm resources for systematic classification and conservation assessment (Daniell et al. 2016; Li et al. 2020a). Therefore, we assembled the complete chloroplast genome sequence of Hopa using the high-throughput Illumina paired-end sequencing data.

## Materials and methods

A single individual Hopa growing at the China National Botanical Garden, Beijing, China (E116.21, N40.01) was used for chloroplast (cp) genome sequencing. A specimen was deposited in the herbarium at Institute of Botany, China National Botanical Garden (Jian Quan, 27255460@qq.com) under the voucher number SQSQJ0082. Total genomic DNA was extracted from young leaves using the DNeasy Plant Mini Kit (Qiagen, Venlo, Netherlands). The paired-end (PE) genomic library was constructed and sequenced on Illumina HiSeq 2500 platform (Illumina, San Diego, CA). Raw paired-end reads were filtered and trimmed using the Fastp program (Chen et al. 2018). The complete cp genome was *de novo* assembled using GetOrganelle (Jin et al. 2020) based on the obtained high-quality PE reads. The Illumina short reads were mapped to the chloroplast genome sequences using BWA v0.7.17 (Li and Durbin 2009) and the coverage depth was calculated using samtools v1.6 (Li et al. 2009). Genome annotation was performed using CPGAVAS2 (Shi et al. 2019). Genome plotting was performed using Chloroplot (Zheng et al. 2020). Map of cis/trans-splicing gene and plastome using CPGView program (http://www.1kmpg.cn/cpgview) (Liu et al. 2023).

We constructed a phylogenetic tree based on the cp genome of Hopa and 30 other species of the genus *Malus*, using *Crataegus hupehensis* Sarg. (Rosaceae: Maloideae) as an outgroup. Sequence alignments were created using MAFFT v7.505 (Nakamura et al. 2018). A maximum-likelihood phylogenetic tree was constructed based on the alignment using IQ-TREE v2.2.0.3 (Minh et al. 2020) with the GTR + I + G model and 1000 bootstraps.

## Results

**Figure 2.**
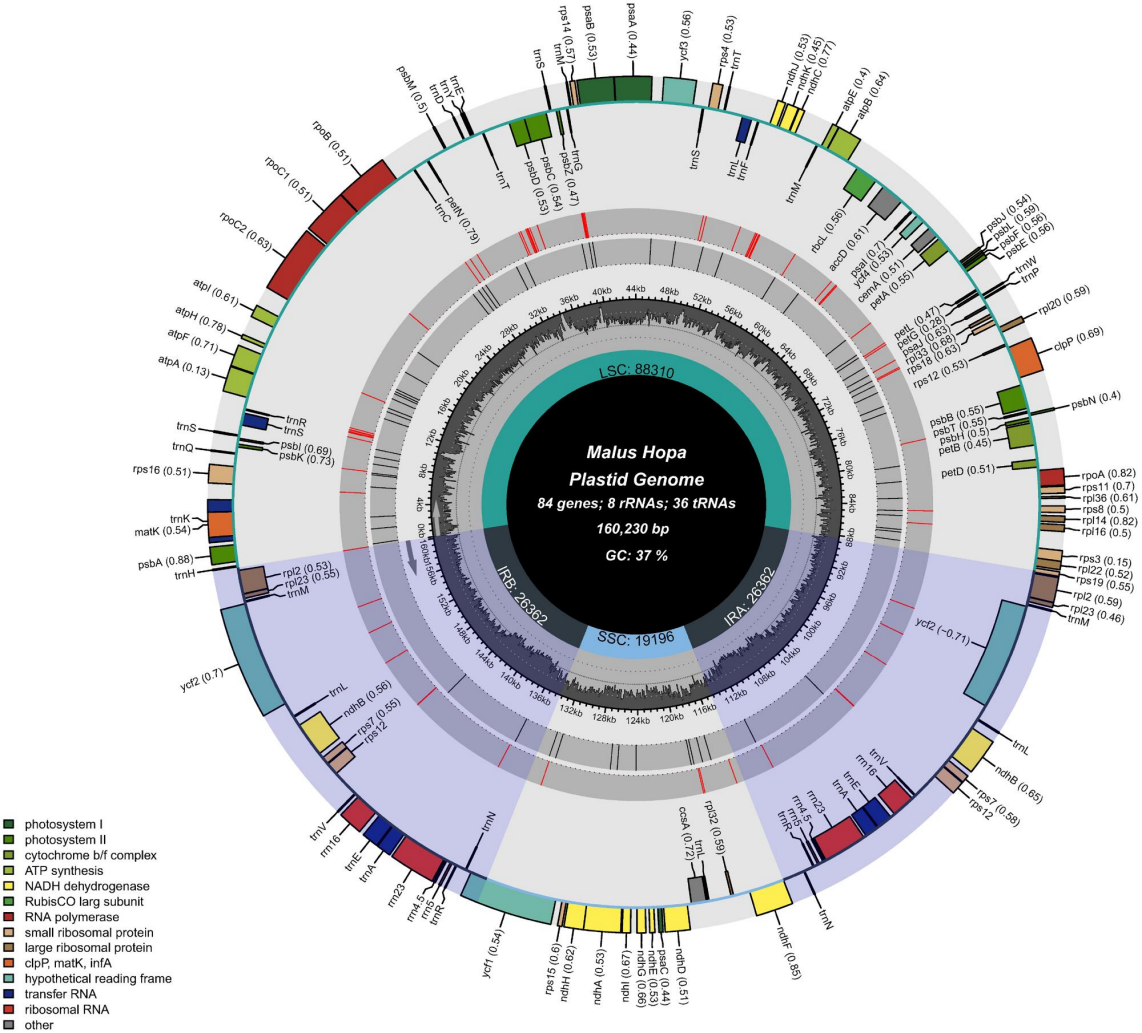
Gene map of the chloroplast genome of Malus × adstringens ‘Hopa’. Circles from innermost to outermost are: the main parts of the chloroplast genome (LSC, SSC, IRA, and IRB); at (light grey) and GC (dark grey) content; SSR density (black); tandem repeat density (red); genes transcribed counter-clockwise (inside) and genes transcribed clockwise (outside). Numbers in parentheses after the gene names indicate the condon usage bias metric. Genes belonging to different functional groups are color-coded. The different colored legends in the lower left corner indicate genes with different functions.

The characteristics of the Hopa cp genome are shown in Figure 2. The total length of the cp genome was 160,230 base pairs (bp) with a GC content of 36.50% and an average coverage of 7557.8-fold (Figure S1), consisting of a large single-copy (LSC) region with a sequence length of 88,310 bp, a small single-copy (SSC) region with a sequence length of 19,196 bp, and a pair of inverted repeat (IR) regions of 26,362 bp. The genome encodes 128 functional genes, including 84 protein-coding genes, 36 tRNA genes (tRNA), and 8 rRNA genes (rRNA). There are 17 genes duplicated in the IR regions, including 6 protein-coding genes (*ndhB*, *rpl2*, *rpl23*, *ycf2*, *rps7*, *rps12*), 7 tRNA genes (*trnA*, *trnE*, *trnL*, *trnM*, *trnN*, *trnR*, *trnV*), and 4 rRNA genes (*rrn4.5*, *rrn5*, *rrn16*, *rrn23*). Totally, 11 cis-splicing genes including *rps16*, *atpF*, *rpoC1*, *ycf3*, *clpP*, *petB*, *petD*, *rpl16*, *rpl2*, *ndhB*, and *ndhA* (Figure S2A), and one trans-splicing genes *rps12* (Figure S2B) were detected. In addition, 82 tandem repeats and 64 SSRs (the search criteria was set as 10 repeats for mononucleotide, 6 for dinucleotide, 5 for trinucleotide, tetranucleotide, pentanucleotide and hexanucleotide repeats) were found in the genome (Table S1, S2).

**Figure 3.**
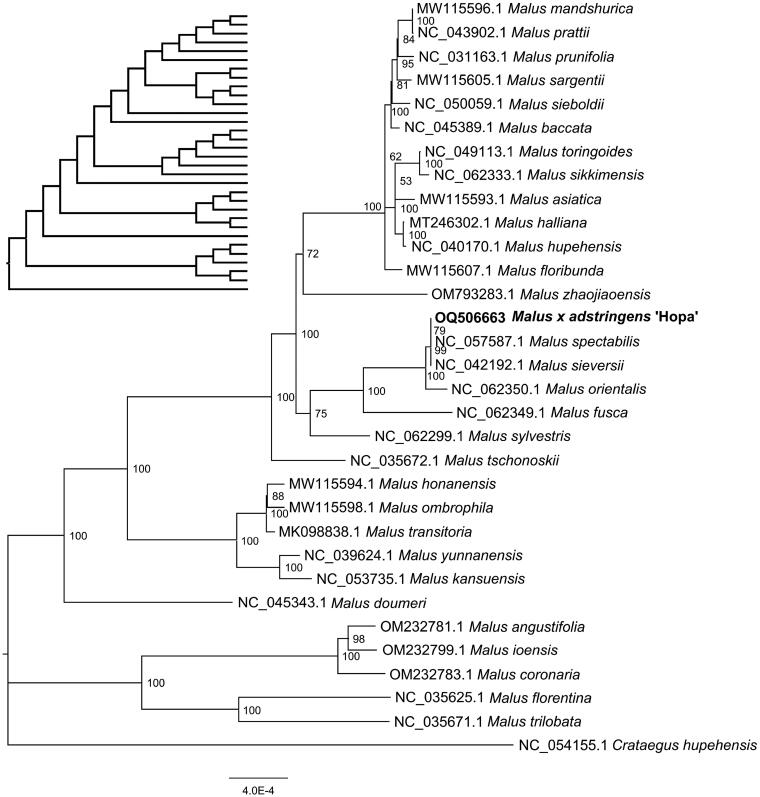
The phylogenetic position for Malus × adstringens ‘Hopa’ according to the maximum likelihood (ML) tree of Malus inferred from 32 chloroplast genomes. The following sequences with GenBank accession were used: Crataegus hupehensis NC_054155.1 (Hu et al. 2021) was used as an outgroup, Malus transitoria MK098838.1 (Zhang et al. 2019), Malus halliana MT246302.1 (Wang et al. 2020), Malus asiatica MW115593.1, Malus honanensis MW115594.1, Malus mandshurica MW115596.1, Malus ombrophila MW115598.1, Malus sargentii MW115605.1, Malus floribunda MW115607.1, Malus prunifolia NC_031163.1 (Bao et al. 2016), Malus florentina NC_035625.1, Malus trilobata NC_035671.1, Malus tschonoskii NC_035672.1, Malus yunnanensis NC_039624.1 (Zeng et al. 2018), Malus hupehensis NC_040170.1 (Zhang et al. 2018), Malus sieversii NC_042192.1 (Naizaier et al. 2019), Malus prattii NC_043902.1 (Fan et al. 2019), Malus doumeri NC_045343.1 (Liu et al. 2020), Malus baccata NC_045389.1 (Liu et al. 2020), Malus bhutanica NC_049113.1 (Li et al. 2020a), Malus toringo NC_050059.1 (Li et al. 2020b), Malus kansuensis NC_053735.1 (Li et al. 2021), Malus spectabilis NC_057587.1 (Jiao et al. 2020), Malus sylvestris NC_062299.1, Malus sikkimensis NC_062333.1, Malus fusca NC_062349.1, Malus orientalis NC_062350.1, Malus angustifolia OM232781.1, Malus coronaria OM232783.1, Malus ioensis OM232799.1 (Liu et al. 2022), Malus zhaojiaoensis OM793283.1 (Wang et al. 2022). Numbers at nodes correspond to ML bootstrap values. The scale bar represents the mean number of nucleotide acid substitutions per site. Cladogram without species names placed top left.

The phylogenetic tree showed that relationships within the genus *Malus* can be well distinguished using the cp genomic data (Figure 3). Hopa was most closely related to *M. spectabilis* (Aiton) Borkh. and then to *M. sieversii* (Lebed.) M. Roem., indicating that they share a common maternal ancestor. These results would be useful for inferring the possible maternal relationships of cultivars of crabapple and apple.

## Discussion and conclusion

The complete chloroplast genome sequence of *Malus* × *adstringens* ‘Hopa’ was first sequenced and found to exhibit a total length of 160,230 bp. A total of 128 genes were annotated, including 84 protein-encoding genes, 36 tRNA genes, and 8 rRNA genes. These features are not significantly different from those of most chloroplast genomes in *Malus* (e.g. Li et al. 2020a, 2020b, Li et al. 2021). The inferred phylogenetic tree analysis confirmed that the phylogenetic relationship of *M*. ‘Hopa’ in *Malus* is closely related to *M. spectabilis* (Aiton) Borkh. and then to *M. sieversii* (Lebed.) M. Roem. The close relationship between *M. spectabilis* and *M. sieversii* was confirmed in a previous study (Jiao et al. 2020). Our results demonstrate that it is feasible to resolve the phylogenetic relationships of crabapple cultivars and identify their putative maternal lineages using chloroplast genomic data.

## Supplementary Material

Supplemental Material

Supplemental Material

## Data Availability

The genome sequence data that support the findings of this study are openly available in GenBank of NCBI at (https://www.ncbi.nlm.nih.gov/) under the accession no. OQ506663. The associated BioProject, SRA, and Bio-Sample numbers are PRJNA954451, SRR24135614, and SAMN34145788, respectively.
