# Supplementary material for: The complete chloroplast genome sequence of Malus × adstringens Zabel ‘Hopa’ (Rosaceae)
Source: Mitochondrial DNA B Resour. 2024 Jan 25;9(1):173–7. doi: 10.1080/23802359.2023.2292158 (PMC10812852; doi:10.1080/23802359.2023.2292158)
Supplement: Supplemental Material [file TMDN_A_2292158_SM5267.doc]

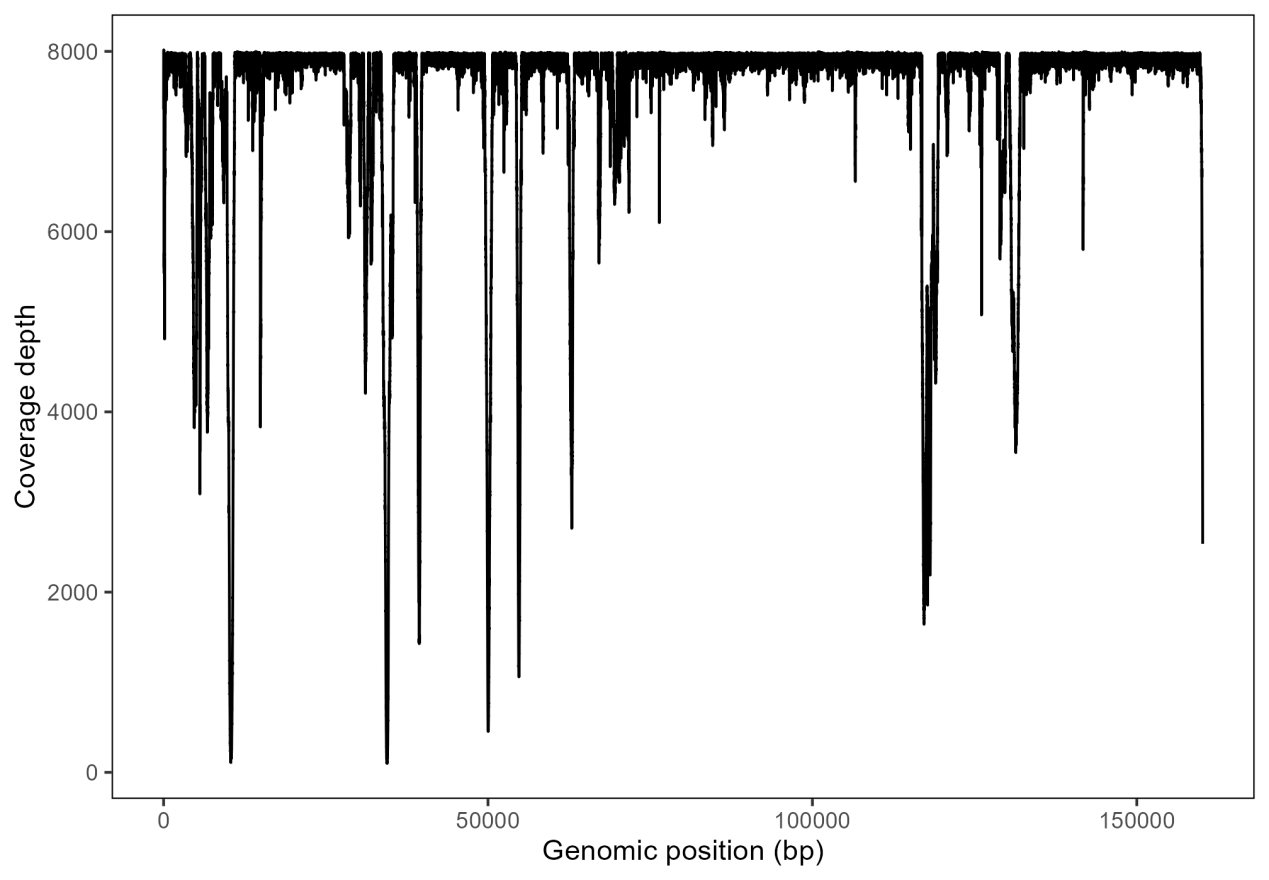


Figure S1. Overall coverage depth of the chloroplast genome assembly of Hopa. The horizontal coordinate indicates the chloroplast length and the vertical coordinate is the coverage depth.

| A | 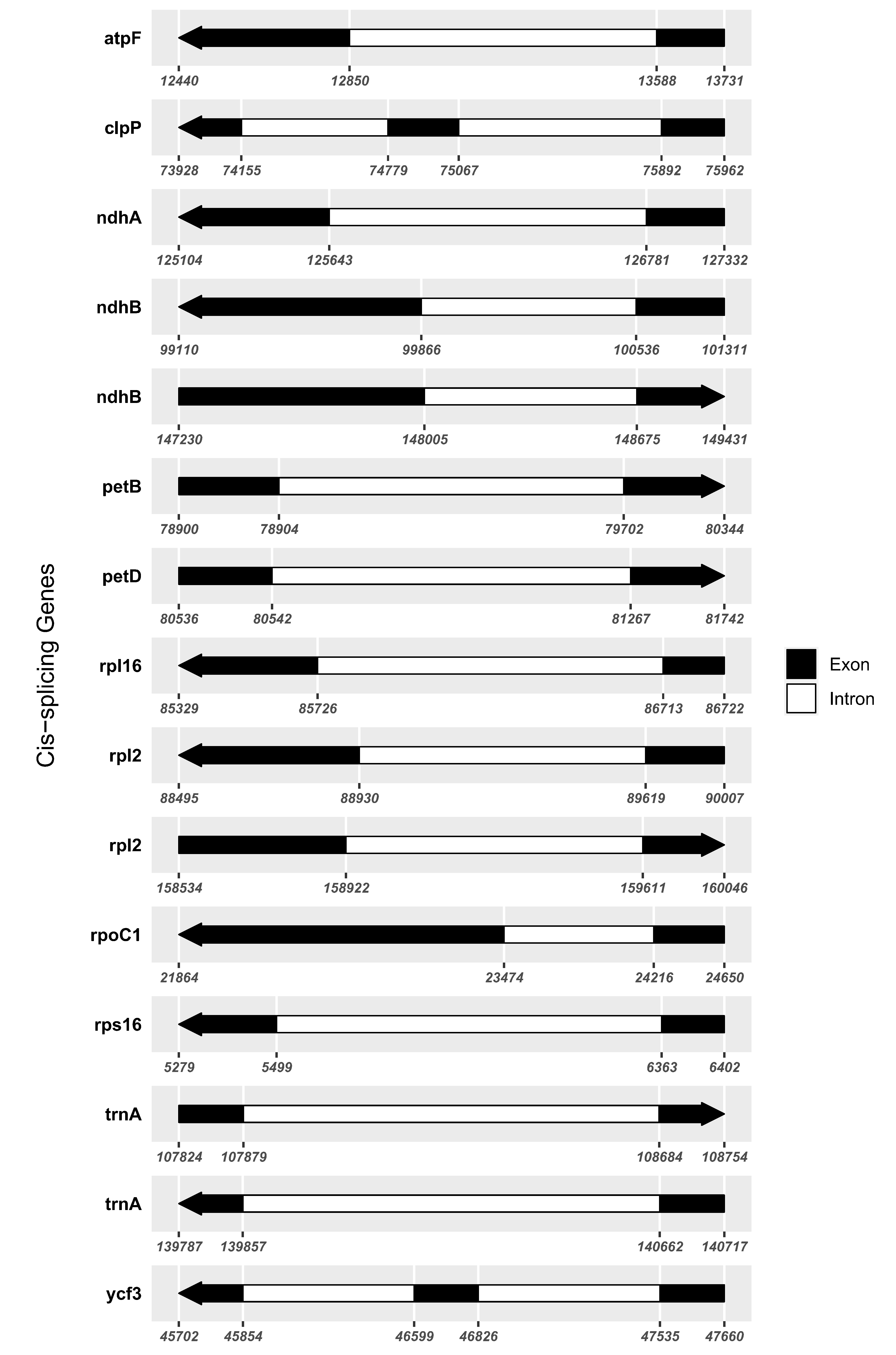 |
| --- | --- |
| B | 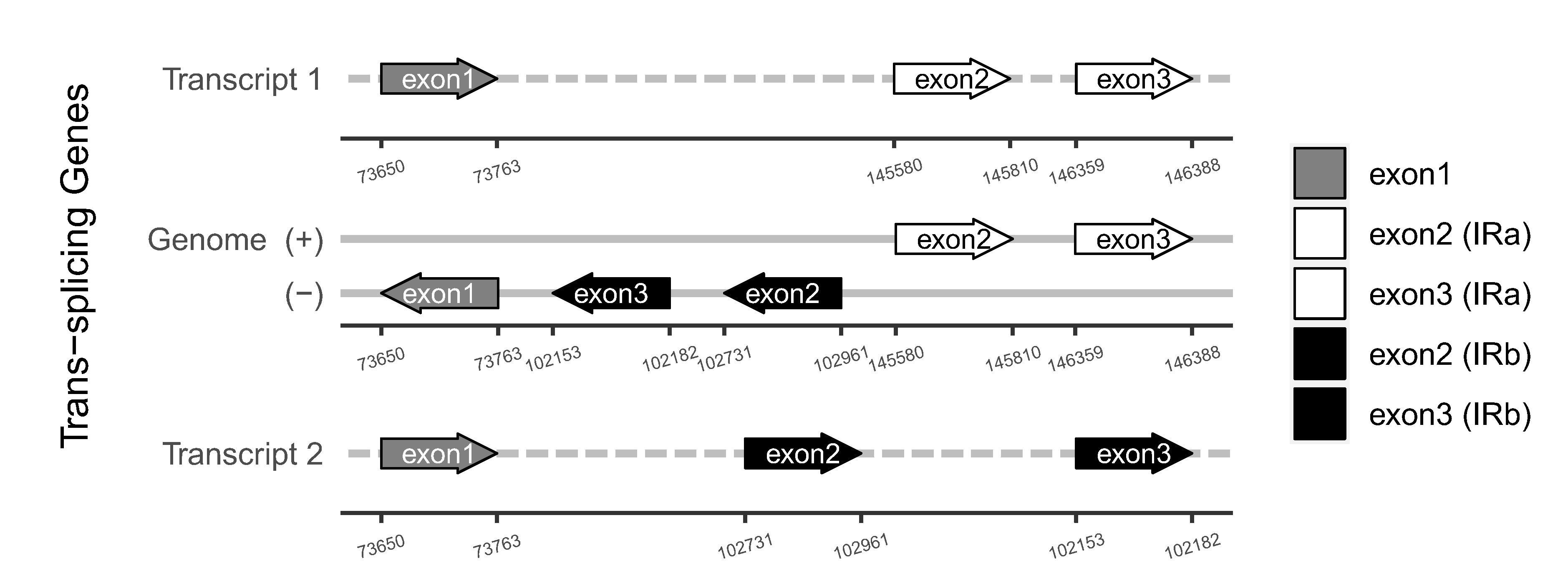 |

Figure S2. Schematic map of the cis-splicing genes (A) and trans-splicing gene rps12 (B) in the Hopa chloroplast genome. The exons and introns are shown in black and white, respectively. Arrows indicate the sense direction of the genes. The map was generated with CPGview.
